# Relationship between progesterone level on the day of human chorionic gonadotropin administration with outcomes of intra-cytoplasmic sperm injection in infertile couples 

**Published:** 2015-07

**Authors:** Mahsomeh Hajishafiha, Zahra Shahbazi, AbdolGhader Pakniyat, Sima Oshnouei, Nazila Kiarang

**Affiliations:** 1 *Reproductive Health Research Center, Gynecology Department, Urmia University of Medical Sciences, Urmia, Iran.*; 2 *Student Research Committee, Emergency Medicine Department, Arak University of Medical Science, Arak, Iran.*

**Keywords:** *Human chorionic gonadotropin*, *Progesterone*, *Intra*-*cytoplasmic sperm injection*, *Pregnancy*, *Infertility*

## Abstract

**Background:**

Gonadotropin-releasing hormone agonists or antagonists are used in assisted reproductive technique cycles as premature luteinizing hormone secretion inhibition. Studies have been reported different and contradictory results on the serum progesterone effect on intra-cytoplasmic sperm injection.

**Objective:**

The purpose of this study was to evaluate the effect of serum progesterone level on the day of Human chorionic gonadotropin (HCG) administration on the intra-cytoplasmic sperm injection (ICSI) outcome in infertile women.

**Materials and Methods:**

249 infertile couples candidated for ICSI were enrolled in the study. Their serum progesterone level on the day of HCG administration was measured and according to serum level, patients were divided into four groups of less than 0.9, 0.9-1.4, 1.5-1.9, and ≥2 ng/mL. The four groups were compared with each other regarding fertility outcomes.

**Results:**

Pregnancy rate was not significantly different among the four groups (p>0.05). Also, there was no significant difference among the groups regarding frequency of abortion and ectopic pregnancy.

**Conclusion:**

Serum progesterone level on the day of HCG administration does not have any significant effect on pregnancy outcomes, including abortion, ectopic pregnancy, and pregnancy rate in patients undergoing ICSI treatment.

## Introduction

Assisted reproductive technique (ART) refers to procedures that are associated with the direct manipulation of ovum outside the body (1). The main steps in these methods include ovum stimulation, collection of ovum, insemination; provide the appropriate environment for fertilization and early embryo growth and embryo transfer in to the uterus. Fertility medications are prescribed to control the timing of the ovum ripening and to increase the chance of collecting multiple ovum during one of the woman’s cycles. Human Chorionic Gonadotropin (HCG) is administrated for the ovum maturation and development, following the HCG administration progesterone level increases. (2, 3). Today gonadotropin-releasing hormone (GnRH) agonists or antagonists are used as prevention of early internal luteinizing hormone (LH) secretion due to estradiol effect during ovum stimulation cycle, despite progesterone level raising before HCG administration is observed in these cycles so in cycles that used GnRH agonist, it’s was reported between 5-38% and in the cycles that used GnRH antagonist, it was reported up to 20% (3-5). Increase of progesterone levels before taking HCG is called premature luteinization and progesterone level more than 0.8-2 ng/ml is considered as rising level of progesterone.

The studies have reported different results in these cycles, some showed that progesterone rising on the day of HCG administration, don’t have effect on ART cycles outcomes, while other studies indicate it has negative effect on ART cycles outcomes (5-11, 15). Some studies indicate rising of progesterone results in harmful effects on the development and maturation of ovum or fertilization and initial egg cell division and results in low quality embryo (12). While in other studies increase of progesterone with inappropriate effect of endothelium and endometrial growth led to impairment in embryo implantation but without any impact on ovulation and embryo growth (11, 16).

Since in various studies there have been different and contradictory results on the serum progesterone effect on intra-cytoplasmic sperm injection (ICSI), the present study aimed at finding relationship between serum progesterone level on the day of HCG administration and ICSI outcomes, so if there was negative relationship between serum progesterone and ICSI outcomes, look for appropriate solutions like early use of HCG, embryo freezing and transfer it in the next cycle (17).

## Materials and methods

This cohort study was conducted at the Infertility Department of Kosar, Urmia, Iran from January 2013 to January 2014. 249 infertile couples with different etiology candidate for ICSI, enrolled in this study. patients with age over 42 years, moderate or severe hydrosalpinges, smoking, history of abortion, uterine anomalies, endometriosis, and who had follicle stimulating hormone (FSH) up to 9 /ml were excluded.

The patients were treated with long protocol buserelin (Gonadotropin Releasing Hormone agonist (GnRH), Superfact; Aventis, France) as subcutaneously, from 21st day of previous cycle and after Superfact continued with half dose since the second day of menstrual cycle, then human menopausal gonadotropin (HMG) (Mengon; Ferring GmbH, Germany) or FSH (Gonal-f; Merck Serona, Germany) was administrated. Gonadotropin dosage was determined based on the patient's age, FSH, and estradiol level on the 3” day of menstrual cycle, volume and number of ovarian follicles on the second day of menstrual cycle by ultrasonography.

Serial vaginal ultrasonography used for evaluation of treatment response and gonadotropin dosage was changed frequently so when three or more than three follicles reached diameter of 17 or18 mm; then 5,000-10,000 units HCG (IVF-C; Pregnil, Iran) was injected intramuscular. 34-38 hs later, the patient was subjected to oocyte retrieval. All of surgery routine laboratory tests were done on the day of HCG administration; also serum progesterone level was requested. The liaison progesterone assay is a chemiluminescent immunoassay (clia) to be used with the liaison- analyzer, for the quantitative of progesterone in human serum and plasma. The diasorin liaison progesterone assay measures between 0.4 and 40.0 ng/ml. limitations of the procedure the laboratory temperature at the time of calibration must be between 20^o^C and 26^o^C. Data including age, etiology of infertility, progesterone level on the day of HCG administration, the number and quality of obtained ovum, number and quality of formed embryos, gonadotropin dosage ,the rate of clinical pregnancy according to heartbeat of fetus observed after 7-8^th^ weeks of pregnancy by ultrasonography, abortion and ectopic pregnancy (EP) were recorded in check list.

According to ovum puncture time, embryo transfer would be done at 48 hours to 72 hours later and the embryos were classified qualitatively based on fragmentation and blastomere size.

Regarding progesterone level on the day of HCG administration, patients were divided into four groups with 0.9>, 0.9-1.4, 1.5-1.9 and 2≤ ng/ml, then the variables in each groups were compared. The aim of study was explained to patients and informed consent was obtained from them. Patients' names remained confidential and no additional cost was imposed to the participants. Ethics committee approval was obtained from Urmia university of medical sciences (umsu.rec.1392.30).

**Statistical analysis**

To compare quantitative data, one-way ANOVA test (or if not normal, Kruskal–Wallis used) and in qualitative outcome, ^2^ test was used. If there were any relationships between each variable and the progesterone level on the day of HCG administration, in order to prevent from progesterone effect on the variables, logistic regression test was used in therapeutic (independent) and leaner regression was used in the quantitative independent variables.

## Results

In this study, 249 infertile women with 20-40 years old undergoing ICSI treatment entered to the study. No significant difference was found between groups regarding age (p=0.51), infertility etiology (p=0.34). FSH of the third day of cycle (p=0.42) and number of used ampoules (p=0.57) in all groups were the same (Table I).

89 pregnancies, 10 abortions and 4 EPs were obtained. 246 cases including 127 cases due to male factors, 94 cases unexplained intrauterine insemination (IUI) (who had 3-4 failed time Intrauterine Insemination), 14 cases ovulation disorder and 11 cases tubal disorder. No significant differences were observed regarding to infertility etiology between the couples based on progesterone level on the day of HCG administration (p=0.34) (Table I).

No Significant difference was observed between the mean number of obtained ovum, ovum quality, number of formed embryo and quality of embryo based on different progesterone level on the day of HCG administration (Table II). There was no relationship between progesterone level on the day of HCG administration and pregnancy events (p=0.89) (Table III). In terms of rates of pregnancy, abortion and EP at different progesterone level on the day of HCG administration, no significant difference was observed (Table III).

**Table I:** Maternal age, Gonadotropin used ampule, and infertility etiology based on progesterone level on the day of HCG administration

**Progesterone level (ng/ml)**		**0.9>**	**0.9-1.4**	**1.5-1.9**	**2<**	**p-value** ^***^
Age (year)*		30.78 ± 5.08	30.55 ± 5.29	29.25 ± 5.58	30.55 ± 5.22	0.51
Gonadotropin Ampule(n) *		33.94 ± 20.84	33.22 ± 21.50	32.30 ± 18.55	27.98 ± 14.69	0.57
FSH level on third day (ng/ml) *		6.42 ± 2.28	7.78 ± 4.59	7.77 ± 7.98	8.75 ± 10.67	0.42
Etiology of infertility**						
	Male factor (n)	30 (51.7)	27 (49.1)	21 (52.5)	49 (52.7)	0.34
	unexplained IUI (n)	18 (31)	20 (36.4)	18 (45)	38 (40.9)	
	Ovulation disorder (n)	5 (8.6)	5 (9.1)	0 (0)	4 (4.3)	
	Tubal and para tubal (n)	5 (8.6)	3 (5.5)	1 (2.5)	2 (2.2)	

HCG: Human chorionic gonadotropin

FSH: Follicle stimulating hormone

IUI: Intrauterine insemination

* Data are presented as mean±SD.

** Data are presented as n (%).

*** Fisher exact test.

**Table II:** Mean number of embryo (MNE),mean number of embryos with B, A quality (MNE-A,B), mean number of obtained ovum (MNO) and mean number of obtained ovum with methaphase-2 (MNO-MT2) based on progesterone level on the day of HCG administration

**Progesterone level (ng/ml)**	**0.9>**	**0.9-1.4**	**1.5-1.9**	**2<**	**p-value** *
MNO	6.5 ± 3.68	6.62 ± 4.02	7.97 ± 4.27	7.83 ± 4.63	0.179
MNO-MT2	6.23 ± 3.61	6.32 ± 4.06	7.03 ± 3.4	7.68 ± 4.52	0.6
MNE	3.90 ± 1.98	4.04 ± 2.36	4.57 ± 2.39	4.70 ± 2.37	0.176
MNE-A,B	4 ± 1.29	4.04 ± 1.42	4.93 ± 1.38	4.92 ± 2.03	0.12

Data are presented as mean±SD.

MNE: Mean number of embryo

MNE-A, B: Mean number of embryos with B, A quality MNO: Mean number of obtained ovum

MNO-MT2: Mean number of obtained ovum with methaphase-2

HCG: Human chorionic gonadotropin

* Kruskal-Wallis test based

**Table III:** Pregnancy rate, number of Abortions and number of EP based on progesterone level on the day of HCG administration

**Progesterone level (ng/ml)**	**0.9>**	**0.9-1.4**	**1.5-1.9**	**2** **≤**	p-value*
Non pregnant (n)	35	33	21	56	0.89
Pregnant	19	19	15	36	
Not aborted	17	16	15	27	0.67
Aborted	1	3	0	6	
Pregnancy	19	19	15	36	0.68
Ectopie Pregrancy	1	0	0	3	

Based Fisher exact test

EP: Ectopic pregnancy

HCG: Human chorionic gonadotropin

## Discussion

GnRH agonists or antagonists are used in ART cycles as premature LH secretion inhibition that it is due to high rising estradiol effects, however progesterone rising before HCG administration is not uncommon; in the patient whom use GnRH agonist it’s frequency has been reported as 5-38% and in the patients that GnRH antagonist was administrated it was reported up to 20% (3-5). Several reasons have been reported for this increase in the literature (4-6). But the more important question is if this increased level on the day that HCG are used, does it have any effect on pregnancy rate, abortion and the incidence of EP in ART cycles or not? Although several studies have been conducted in this topic and each have obtained different results, the role of premature progesterone rising has remained obscure (5, 10, 12, 15, 18).

Rehana Rehman et al indicated that a high estradiol/progesterone ratio on the day of ovulation induction predicts the success of intra cytoplasmic sperm injection (19).

Even studies that indicate that progesterone has harmful side effects on ART cycles, but they are not agree with the mechanism, so some studies indicated that progesterone on the day of HCG administration, has harmful side effects on development and final maturation of ovum, fertilization, and initial egg cell division and low quality of embryo; and some others consider its inappropriate effect as a result of its impact on endothelium and endometrial growth which leads embryo implantation failure (11, 12, 16).

In the study by Abuzeid *et al.* progesterone greater than 0.9 ng/ml was associated with more matured follicles and more oocytes, however like the current study there were no differences between groups regarding fertilization rate and the embryo formation (6). Other findings were similar to the results of the study by Huang *et al* (7, 11, 16). In the study by Yovel et al, high levels of progesterone were associated with oocytes or embryos quality disorders (12). In the study of Miloy et aL, high levels of progesterone were associated with a greater amount of oocytes; however the number of mature oocytes and fertilization in this cycle was less (13). In the study of Bustillom et al, increased progesterone level on the day of HCG administration, was associated with increased number of oocytes (5).

There was no significant difference between groups regarding pregnancy rate, based to progesterone level on the day of HCG administration, (p=0.98) which indicates no effect of progesterone level on the day of HCG administration, on the endothelium and endometrial growth. Other findings were similar to the results of other studies that showed premature progesterone rising has no effect on pregnancy rate (5, 7, 9, 11, 16). The study by Abuzied et al revealed that premature progesterone rising has no effect on clinical pregnancy rate, abortion and live births rate (6). 

Moffitt *et al*. showed that progesterone level on the day of HCG administration is not a suitable marker for embryo transfer cancellation in fresh and freeze cycles (8). In contrast to these findings, others pointed at low pregnancy rate due to progesterone levels rising on the day of HCG administration (12-15). In the study by Papanikolaou et al, the middle progesterone rising had a negative effect on embryo implantation with good quality (20).

## Conclusion

Regarding the incidence of abortion in our study, there was no statistically significant difference between four groups with different progesterone level on the day of HCG administration (p=0.28); which at is line with other studies (6, 9-11). Regarding the rate of EP in our study, no significant difference was found between the levels of progesterone level on the day of HCG administration and EP (p=0.68), but in the group that had progesterone levels ≥2, the incidence of EP was more other groups (p=0.08). In other studies, the rate of EP at different progesterone level on the day of HCG administration was not investigated, which can be due to different methods of progesterone level classification.

According to the results of the present study, progesterone level on the day of HCG administration does not have any effect on pregnancy rate, abortion and EP in patients undergoing ICSI treatment. To reach decisive and final result, further and broader studies with same classified progesterone level in this area should be conducted.

## Note

***This article extracted from M.D. thesis. (Zahra Shahbazi)***
